# Who becomes a dermatologist? A repeated cross-sectional study on diversity in the Dutch dermatology workforce

**DOI:** 10.1371/journal.pone.0350963

**Published:** 2026-06-12

**Authors:** Sheeney T. Magdalena, Angelo Li, Jim E. Zeegelaar, Lianne Mulder

**Affiliations:** 1 Faculty of Medicine, Vrije Universiteit Amsterdam, Amsterdam, the Netherlands; 2 Faculty Health Policy and Management, Erasmus University, Rotterdam, the Netherlands; 3 Department of Dermatology, Amsterdam UMC, Amsterdam, the Netherlands; 4 Department of Dermatology, Flevoziekenhuis, Almere, the Netherlands; 5 Department of Global Public Health and Bioethics, Julius Center for Health Sciences and Primary Care, University Medical Center Utrecht, Netherlands; Faculty of Medical Sciences of Minas Gerais, BRAZIL

## Abstract

**Background:**

Workforce diversity in dermatology is crucial for equitable, high-quality care, given the impact of skin tone, culture, and socio-economic status on skin conditions. Although this has been studied in other countries, data on the demographic makeup of Dutch dermatologists is lacking. This study aims to assess workforce diversity in Dutch dermatology over time.

**Methods:**

We conducted a nationwide repeated cross-sectional study using pseudonymized microdata from Statistics Netherlands, including sex, migration background, and parental socio-economic indicators. Descriptive statistics were used to track demographic trends between 2005 and 2023, and multivariable logistic regression analyses were performed to evaluate which variables influenced the odds that registered physicians in the 2023 national healthcare professional register (BIG register) had to be a dermatologist.

**Results:**

Female representation rose from 35.4% in 2005 to 61.7% in 2023. In 2023, 84.4% of dermatologists had no migration background or a European migration background. Dermatologists with Turkish, Moroccan, Surinamese, or Caribbean Dutch origins were underrepresented. Despite a net increase of 289 dermatologists, only 56 of this net increase consisted of dermatologists with a non-European background. Multivariable regression analysis showed that being female (OR 1.609 [1.143–2.266]), having parents in the top 20% assets bracket (OR 2.251 [1.272–3.984]), or having physician parents (OR 1.326 [1.011–1.740]) were associated with higher odds of being a registered dermatologist among the younger generation of physicians.

**Conclusions:**

The findings highlight a persistent lack of ethnic and socio-economic diversity in the Dutch dermatology workforce, despite broader demographic shifts in the general population and medical student cohorts. The underrepresentation of dermatologists with a migration background may have implications for equitable patient care, particularly in the context of cultural and linguistic barriers, as well as differences in disease presentation across skin tones. Further research is warranted to explore the potential impact of workforce diversity on patient outcomes.

## Introduction

Diversity in healthcare is increasingly recognized as essential for delivering high-quality, patient-centered care, particularly in specialties like dermatology where conditions manifest differently across skin tones and ethnic groups [1]. Much of the existing research on workforce diversity in dermatology has been performed in the United States [2–7]. These studies highlight significant underrepresentation of individuals from minority backgrounds in the dermatology workforce, often pointing to barriers at multiple stages of medical education and career advancement [8,9]. This can affect both patient care and workforce development: In dermatology, where common skin conditions and hair disorders manifest differently across skin types and different ethnic groups, a lack of diversity in the workforce can result in misdiagnosis and suboptimal care for minority patients [10–16]. Patients from ethnic minority backgrounds often report receiving better care from minority physicians who understand their cultural and dermatological needs [8,17,18]. This concordance has been shown to improve communication, trust, and treatment outcomes [8,19]. Additionally, a more diverse workforce can contribute to reducing healthcare disparities by fostering cultural competence among all healthcare providers [6,8].

There is a notable lack of comprehensive data on how the diversity of the dermatology workforce in the Netherlands has developed over time. Dutch research has shown that the shift from lottery-based to selection-based admissions in Dutch medical education has reduced student diversity and reinforced barriers for ethnic minority applicants [20]. Additionally, physicians with a migration background, especially women, remain underrepresented in medical specialties, raising concerns about whether dermatology reflects the population’s growing diversity [21,22].

In the United States, limited workforce diversity and training gaps contribute to delayed diagnoses in minority patients [9,10]. In the Netherlands, no data exist on misdiagnoses or diagnosis delays linked to skin tone. Still, the underrepresentation of ethnic minorities among specialists and the lack of darker skin tones in training materials suggest a risk of misdiagnosis or delayed diagnosis, though the true impact remains unknown [23,24].

In the Netherlands, it is unclear how workforce diversity in dermatology affects access and career progression. This highlights the need to investigate the dermatology workforce to ensure it meets the needs of an increasingly diverse patient population. Our study aims to fill this gap and inform more inclusive healthcare policies and recruitment practices, ultimately enhancing the quality of dermatological care.

This study investigates diversity within the Dutch dermatology workforce, with three objectives: (1) to assess diversity by sex, migration background, socio-economic status, and having healthcare professional parents; (2) to analyze changes between 2005 and 2023; and (3) to evaluate whether demographic background characteristics influence the odds of becoming a dermatologist.

## Methods

### Study design

To meet objective 1 and 2, we conducted a repeated cross-sectional study of the healthcare professional register (hereafter: ‘BIG register’) using anonymized non-public microdata from Statistics Netherlands, following the protocol in S1 Protocol. We used the STROBE Statement [25] as our guideline. To meet objective 3, we used logistic regression analyses, performed on the physicians with an active registration in the BIG-register of 2023.

### Study population

Within the BIG-register, we determined which physicians had an active registration (on at least one day) in the years 2005, 2010, 2015, 2020 and 2023, and whether they were also a registered dermatologist. A BIG registration is legally required in order to practice as a physician and dermatologist. There were no exclusion criteria.

### Variables

Variables in the study are described in Table 1. The outcome measure of the regression analyses was: is the physician also a registered dermatologist (no/yes).

**Table 1:** Variables in the study.

Variable	Values	Rationale for categorization	Rationale for selecting variable
Sex	0 = Male, 1 = Female		The known male:female ratios of physicians and dermatologists in 2021 [24].
Migration background	Statistics Netherlands categorizes migration background based on someone’s country of birth, and/or that of their parents. If one parent was born in the Netherlands and the other abroad, the country other than the Netherlands is chosen. We recoded all country codes in the following groups: 0 = No migration background, 1 = Europe (excl. The Netherlands), 2 = Turkey, 3 = Morocco, 4 = Suriname, 5 = Dutch Caribbean, 6 = Indonesia, 7 = Other Africa, 8 = Other Asia, 9 = Other America and Oceania. Due to small sizes of certain groups, we combined several migration backgrounds in a number of regression analyses. The descriptive statistics are, wherever permitted within the privacy regulations around group sizes, given in as much detail as possible to enable better interpretation.	The differences in types of migration history of migrant populations.	The known inequalities which healthcare professionals with a migration background face in the selection procedures for residency training [26] and in the healthcare sector in general [28,29].
Year of birth	Categorized for regression analysis: 0 = Year of birth 1979 or earlier, 1 = Year of birth 1980 or later	Best possible balance in each category amongst physicians	
**Parental data**			
Assets percentile	Scale of 0–100, categorized for regression analysis: 0 = Percentiles 1–60, 1 = Percentiles 61–80, 2 = Percentiles 81–100	Best possible balance in each category	The disproportionate share of physicians from high-SES families among physicians [24]. Assets percentiles, rather than their value in euros, were included because percentiles indicate the relative position one occupies compared to the rest of the population.
Number of parents who receive social welfare	0, 1, 2, categorized for regression analysis: 0 = 0 parents, 1 = 1 or 2 parents		The disproportionate share of physicians from high-SES families among physicians [24].
Number of parents who are registered healthcare professionals	0, 1, 2, categorized for regression analysis: 0 = 0 parents, 1 = 1 or 2 parents		The known influence of having a network in the medical field as a facilitator in the route from childhood to practising medicine [24]
Number of parents who are also physicians	0, 1, 2, categorized for regression analysis: 0 = 0 parents, 1 = 1 or 2 parents		The known influence of having a network in the medical field as a facilitator in the route from childhood to practising medicine [24].

### Bias

We followed Šimundić’s [26] classifications of bias in research to study potential sources of bias in advance of the analyses. By including *all* physicians and dermatologists in 5 different years, we aimed to eliminate potential bias in sampling. The size of the total population sample in each year can be found in Table 2.

**Table 2:** Demographic statistics of physicians and Dermatologists, in different years and age categories.

	DERMATOLOGISTS														PHYSICIANS					
	BIG – 2023		BIG – 2020		BIG – 2015		BIG – 2010		BIG – 2005		BIG – 2023: born ≤1979		BIG – 2023: born ≥1980		BIG – 2023: all ages		BIG – 2023: born ≤1979		BIG – 2023: born ≥1980	
	Freq	%	Freq	%	Freq	%	Freq	%	Freq	%	Freq	%	Freq	%	Freq	%	Freq	%	Freq	%
Total	682	100	666	100	573	100	496	100	393	100	418	100	264	100	79537	100	37602	100	41935	100
**SEX**																				
Male	261	38.3	274	41.1	283	49.4	275	55.4	254	64.6	194	46.4	67	25.4	33267	41.8	19980	53.1	13287	31.7
Female	421	61.7	392	58.9	290	50.6	221	44.6	139	35.4	224	53.6	197	74.6	46270	58.2	17622	46.9	28648	68.3
MIGRATION BACKGROUND*																				
None	503	73.8	492	73.9	414	72.3	358	72.2	296	75.3	305	73	198	75	60858	76.5	28724	76.4	32134	76.6
Europe (excl· NL)	75	11	74	11.1	73	12.7	67	13.5	49	12.5	51	12.2	24	9.1	6336	8	3146	8.4	3190	7.6
Turkey	<5		<5		<5		<5		<5		<5		<5		937	1.2	303	0.8	634	1.5
Morocco	<5		<5		<5		<5		<5		<5		<5		497	0.6	91	0.2	406	1
Suriname	16	2.3	16	2.4	12	2.1	11	2.2	<10		<10		<10		1679	2.1	859	2.3	820	2
Dutch Caribbean	13	1.9	13	2	10	1.7	<10		<5		<10		<10		935	1.2	368	1	567	1.4
Indonesia	41	6	42	6.3	41	7.2	38	7.7	32	8.1	31	7.4	10	3.8	3370	4.2	2427	6.5	943	2.2
Other Africa	<5		<5		<5		<5		<5		<5		<5		814	1	270	0.7	544	1.3
Other Asia	19	2.8	17	2.6	13	2.3	<10		<5		<10		<15		3085	3.9	1088	2.9	1997	4.8
Other America and Oceania	<10		<10		<10		<10		<5		<10		<5		1021	1.3	321	0.9	700	1.7
Missing															5	0	5	0		
INTERSECTIONAL IDENTITIES*																				
Man without MB	181	26.5	191	28.7	200	34.9	204	41.1	196	49.9	137	32.8	44	16.7	24862	31.3	15068	40.1	9794	23.4
Woman without MB	322	47.2	301	45.2	214	37.3	154	31	100	25.4	168	40.2	154	58.3	35996	45.3	13656	36.3	22340	53.3
Man with European MB	28	4.1	29	4.4	33	5.8	26	5.2	25	6.4	<25		<10		2630	3.3	1555	4.1	1075	2.6
Woman with European MB	47	6.9	45	6.8	40	7	41	8.3	24	6.1	<30		<20		3706	4.7	1591	4.2	2115	5
Man with Tur/Mar MB	<5		<5		<5		<5		<5		<5		<5		617	0.8	223	0.6	394	0.9
Woman with Tur/Mar MB	<5		<5		<5		<5		<5		<5		<5		817	1	171	0.5	646	1.5
Man with Sur/Car MB	15	2.2	17	2.6	<15		11	2.2	<10		<10		<10		1134	1.4	680	1.8	454	1.1
Woman with Sur/Car MB	14	2.1	12	1.8	<10		<10		<5		<10		<10		1480	1.9	547	1.5	933	2.2
Man with Indonesian MB	<25		<25		25	4.4	26	5.2	22	5.6	<20		<5		1827	2.3	1484	3.9	343	0.8
Woman with Indonesian MB	<20		<20		16	2.8	12	2.4	10	2.5	<15		<10		1543	1.9	943	2.5	600	1.4
Man with Other MB	13	1.9	12	1.8	12	2.1	<10		<5		<10		<5		2194	2.8	967	2.6	1227	2.9
Woman with Other MB	18	2.6	15	2.3	10	1.7	<10		<5		<10		<15		2726	3.4	712	1.9	2014	4.8
Missing															5	0	5	0		
NUMBER OF PARENTS WHO RECEIVE SOCIAL WELFARE*																				
0	<685		<665		<575		<500								78743	99	37564	99.9	41179	98.2
1 or 2	<5		<5		<5		<5								794	1	38	0.1	756	1.8
HIGHEST ASSETS PERCENTILE OF EITHER PARENT*																				
1-60	37	5.4									23	5.5	14	5.3	5796	7.3	1837	4.9	3959	9.4
61-80	50	7.3									26	6.2	24	9.1	8385	10.5	2533	6.7	5852	14
81-100	274	40.2									125	29.9	149	56.4	27677	34.8	8059	21.4	19618	46.8
Missing	321	47.1									244	58.4	77	29.2	37679	47.4	25173	66.9	12506	29.8
**Number of parents with a BIG-registration**																				
0	525	77	526	79	484	84.5	432	87.1	362	92.1	338	80.9	187	70.8	62705	78.8	32519	86.5	30186	72
1 or 2	157	23	140	21	89	15.5	64	12.9	31	7.9	80	19.1	77	29.2	16832	21.2	5083	13.5	11749	28
**Number of parents who are also a physician**																				
0	583	85.5	577	86.6	509	88.8	449	90.5	365	92.9	359	85.9	224	84.9	71162	89.5	34422	91.5	36740	87.6
1 or 2	99	14.5	89	13.4	64	11.2	47	9.5	28	7.1	59	14.1	40	15.2	8375	10.5	3180	8.5	5195	12.4

MB = migration background

* some data cannot be shown due to CBS privacy regulations around traceability

### Statistical analysis

Descriptive statistics of the variables in Table 1, based on cross-sectional analyses of the BIG-registers of 2005, 2010, 2015, 2020 and 2023, provide the information to assess the (development of the) diversity of the dermatology workforce. Within the BIG-register of 2023, we also created two groups based on year of birth (born in 1979 or earlier; born in 1980 or later), to show the diversity within each age category. For the variable ‘number of parents receiving social welfare’, data were not available for all reference years. Therefore, we used data from the closest available years (2011 for 2010, 2021 for 2023). We analyzed the variable ‘Highest assets percentile of either parent’ using only data of the physicians and specialists registered in 2023, with the parental assets data of 2022 as those were the most recent data available. Because wealth accumulates over time, comparing parental assets across years could reflect changes in parents’ wealth for the same dermatologists, not a change in the socio-economic diversity of the dermatologist workforce itself.

To evaluate the odds of physicians from different demographic groups to be a dermatologist, we performed univariable and multivariable logistic regression analyses on the physicians in the BIG-register of 2023, to examine which demographic variables were associated with being a dermatologist. Statistical level of significance was set at 0.05. We examined data for evidence of multicollinearity amongst the independent variables using both the variance inflation factor (VIF) of each variable and the tolerance values. Multicollinearity was examined based on the total pool of physicians and specialists. Both the univariable and multivariable analyses were performed on the total group of physicians (regardless of age), and on the younger generation of physicians (those born in 1980 or later). As the variables ‘number of parents who are registered healthcare professionals’ and ‘number of parents who are also physicians’ partially overlap (physicians are healthcare professionals, but healthcare professionals are not necessarily physicians), we created two separate multivariable models. Analyses were performed using IBM SPSS software for Windows, Version 25.0 (IBM Corp, Armonk, NY).

### Data sources

The statistical results are based on calculations by LM using non-public anonymized microdata from Statistics Netherlands (project number 9656). The researchers had no access to identifiable information. The authors of the manuscript do not own any raw data, and under Dutch law, it is not legal to publicly share these data. The statistical results comply with all Statistics Netherlands privacy regulations and the Dutch law regarding use of non-public microdata [27]. Under certain conditions, these microdata are accessible for statistical and scientific research. For further information about how to access the raw data, see the Statistics Netherlands website: https://www.cbs.nl/en-gb/our-services/customised-services-microdata/microdata-conducting-your-own-research.

### Data of small groups

Statistics Netherlands prohibits disclosure of group sizes (including within-variable categories) smaller than 10. We therefore replaced frequencies between 0 and 4 by ‘<5’ and frequencies between 5 and 9 by ‘<10’. Regression analyses were performed for all groups, regardless of group sizes, but due to Statistics Netherlands regulations, the regression results of group sizes <5 could not be published.

### Ethics statement

This study does not fall under the scope of the Dutch Medical Research Involving Human Subjects Act (WMO). It therefore does not require approval from an accredited medical ethics committee in the Netherlands. However, in the UMC Utrecht, an independent quality check has been carried out to ensure compliance with legislation and regulations (regarding Informed Consent procedure, data management, privacy aspects and legal aspects).

## Results

### Participants

Table 2 summarizes the demographic data of all physicians in 2023, all dermatologists in the 5 different years, and all physicians and dermatologists in 2023 divided into two age groups.

As of 2023, the dermatology workforce in the Netherlands shows a significant imbalance in terms of sex and migration background. Women make up 61.7% of dermatologists, a proportion that is slightly higher than the overall physician pool, where 57.3% of physicians are female. Ethnic diversity within the dermatology workforce remains critically low. 84.4% of dermatologists had either no migration background (73.2%) or a European migration background (11.2%). In 2005, this percentage was 87.8%, meaning that ethnic diversity has barely increased in 18 years time.

In every year, there were fewer than 5 dermatologists with a parent who received social welfare. When missing data are excluded, the vast majority had parents in the top-20% assets category: 75.9% in the total age group, 71.8% in the older generation and 79.7% in the younger generation.

**Fig 1:**
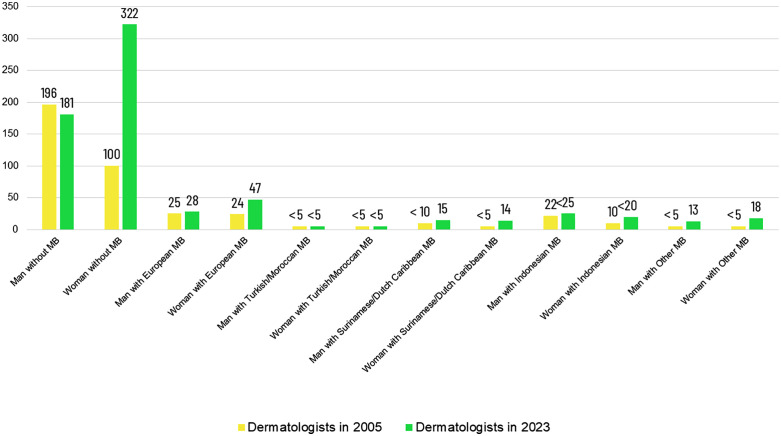
Intersectional identity characteristics (sex + migration background) of Dutch dermatologists in 2005 and 2023.

Between 2005 and 2023, the dermatology workforce saw a net increase of 289 specialists. Out of this net increase, 126 had a health professional parent (43.5%), out of which 71 were also a physician (24.6%). As Fig 1 shows, the majority of the net increase of 289 dermatologists in the BIG-register consists of women without a migration background (+222, 68.9%).

No evidence was found for multicollinearity (the tolerance values for the different models ranged between 0.899–0.995 and the VIF values for the different models ranged between 1.005–1.113). Table 3 contains the univariable logistic regression results for the variables sex, migration background, parental assets percentile, the number of parents who are registered healthcare professionals, and the number of parents who are also physicians. We also created intersectional groups, based on the combination of sex and migration background, to determine whether different demographic groups of physicians had different odds of being a dermatologist. Due to the low numbers of people (<5) in certain intersectional groups, no further division could be made in these intersectional groups using any other additional variable. Table 4 gives the multivariable regression model for the variables sex, migration background, parental assets percentile, and the number of parents who are also physicians. S2 Table contains the multivariable model in which ‘parents who are also physicians’ is replaced by ‘parents who are registered healthcare professionals’.

**Table 3:** Univariable logistic regression.

	Dermatologists, born ≥ 1980			Dermatologists, all ages		
	p-value	Unadjusted odds ratio	95% C.I.	p-value	Unadjusted odds ratio	95% C.I.
**SEX**						
Woman (ref: man)	**0.019**	**1.393**	1.055-1.839	0.077	1.143	0.986-1.326
**MIGRATION BACKGROUND**						
Europe (ref: none)	0.378	1.210	0.791-1.851	**0.001**	**1.476**	1.170-1.863
Non-European (ref: none)	0.894	1.023	0.733-1.428	0.569	1.060	0.866-1.298
**PARENTAL ASSETS PERCENTILE**						
Cat 61–80 (ref. 1–60)	0.567	1.211	0.629-2.333	0.743	0.932	0.611-1.420
Cat 81–100 (ref. 1–60)	**0.005**	**2.179**	1.259-3.772	**0.01**	**1.563**	1.113-2.196
**NUMBER OF PARENTS WHO ARE REGISTERED HEALTHCARE PROFESSIONALS**						
1 or 2 (ref. 0)	0.749	1.044	0.801-1.362	0.503	1.062	0.891-1.266
**NUMBER OF PARENTS WHO ARE ALSO PHYSICIANS**						
1 or 2 (ref. 0)	0.187	1.255	0.895-1.758	**0.003**	**1.384**	1.121-1.709
**INTERSECTIONAL IDENTITY**						
Woman without migration background	**0.008**	**1.575**	1.126-2.203	**0.034**	**1.208**	1.014-1.439
Man with European MB	0.608	1.251	0.532-2.942	0.053	1.455	0.995-2.128
Woman with European MB	**0.020**	**1.918**	1.106-3.326	**<0.001**	**1.803**	1.327-2.450
Man with Turkish/Moroccan MB	*			*		
Woman with Turkish/Moroccan MB	*			*		
Man with Surinamese/Dutch Caribbean MB	**<0.001**	**3.993**	1.869-8.531	**0.006**	**1.965**	1.209-3.194
Woman with Surinamese/Dutch Caribbean MB	0.087	1.937	0.909-4.126	0.329	1.300	0.768-2.202
Man with Indonesian MB	*			**0.007**	**1.759**	1.166-2.653
Woman with Indonesian MB	0.063	2.251	0.955-5.303	**0.04**	**1.624**	1.023-2.579
Man with Other MB	*			0.484	0.824	0.478-1.418
Woman with Other MB	0.532	1.235	0.637-2.396	0.702	0.912	0.569-1.462

* Results could not be published for intersectional groups who consisted of fewer than 5 dermatologists, due to privacy regulations of Statistics Netherlands.

**Table 4:** Multivariable logistic regression.

	Dermatologists, born ≥ 1980			Dermatologists, all ages		
	p-value	Adjusted odds ratio	95% C.I.	p-value	Adjusted odds ratio	95% C.I.
**SEX**						
Woman (ref: man)	**0.006**	**1.609**	1.143-2.266	**0.049**	**1.252**	1.001-1.565
**MIGRATION BACKGROUND**						
Europe (ref: none)	0.111	1.649	0.891-3.053	0.269	1.31	0.812-2.113
Non-European (ref: none)	0.146	1.377	0.894-2.119	0.622	1.086	0.782-1.509
**PARENTAL ASSETS PERCENTILE**						
Cat 61–80 (ref. 1–60)	0.444	1.298	0.666-2.529	0.765	0.937	0.611-1.436
Cat 81–100 (ref. 1–60)	**0.005**	**2.251**	1.272-3.984	**0.025**	**1.498**	1.053-2.131
**NUMBER OF PARENTS WHO ARE ALSO PHYSICIANS**						
1 or 2 (ref. 0)	0.201	1.279	0.877-1.865	**0.042**	**1.326**	1.011-1.740

The univariable results show that amongst the total group of physicians in 2023, those with a European migration background had significantly higher odds of being a dermatologist, just like physicians with parents in the top-20% assets percentile, and with at least one parent who was also a physician. Amongst the younger generation of physicians (born ≥1980), women and physicians with parents in the top-20% assets category had significantly higher odds. The intersectional analyses show that both in the total group of physicians and within the younger generation, there were several groups with higher odds to be a dermatologist, in comparison to male physicians without a migration background: women without a migration background or a European migration background, and men with a Surinamese or Dutch Caribbean background. Amongst the total pool of physicians, this pattern was also found for men and women with an Indonesian background.

The multivariable analysis reveals significant disparities in the odds of becoming a dermatologist based on sex, socio-economic background, and family ties to the medical profession. Among the younger generation of physicians, female physicians have significantly higher odds to be a dermatologist compared to their male counterparts (OR 1.609 [1.143–2.266]). Additionally, physicians whose parents belong to the top 20% assets bracket have higher odds (OR 2.251 [1.272–3.984]). Looking at the total physician pool, female physicians (OR 1.252 [1.001–1.565]), physicians with parents in the top 20% assets bracket (OR 1.498 [1.053–2.131]) and physicians with one or two parents who are physicians (OR 1.326 [1.011–1.740]) also have higher odds to be a dermatologist. S2 Table shows that the effect of having parents who are BIG-registered healthcare professionals (in any profession) was not significant.

## Discussion

### Key findings

Our analysis reveals three major patterns in the Dutch dermatology workforce diversity. First, the majority of dermatologists in 2023 is female (61.7%), which is higher than in the total pool of physicians (57.3% female). The generational sex differences, however, are much larger: amongst the older generation of dermatologists, 53.6% is female, compared to 74.6% of the younger generation. To contextualize this within broader demographic trends: In 2020, the Dutch first year medical student population was 67.6% female [28], suggesting that the increasing female representation in dermatology is even stronger than the overall shift in medical education and the physician workforce. This increase primarily concerned female dermatologists without a migration background, which grew by 22.6 percentage points since 2005.

Second, ethnic diversity remains stagnant despite demographic changes in Dutch society [29]. The percentage of dermatologists with non-European backgrounds increased minimally from 12.2% to 15.6%, while European backgrounds decreased slightly (87.8% to 84.8%). This contrasts sharply with medical student diversity, where 23.6% of first-year students have non-European backgrounds [28]. Specific minority representation shows negligible growth: Turkish, Moroccan, and African backgrounds remain below 5 individuals each; Surinamese and Dutch Caribbean backgrounds increased to only 16 and 13 individuals respectively; Indonesian backgrounds grew from 32 to 41; and other Asian backgrounds from <5–19—all minimal gains despite adding 289 dermatologists overall during this period. These numbers are especially low considering the size of the Dutch population who were born in, or whose parents were born in, Turkey (>457.000), Morocco (>429.000) or another part of Africa (>377.000), Suriname (>365.000), the Dutch Caribbean islands (>194.000), Indonesia (>356.000) or another part of Asia (>848.000), and America and Oceania (>295.000) [29].

Third, socio-economic homogeneity is evident, with 59.3% of younger dermatologists having at least one parent in the top 20% assets bracket (79.7% when missing data are excluded). Our multivariable model shows that being female, having high-asset parents, and physician parents is associated with higher odds of being a registered dermatologist.

The underrepresentation of key minority groups is notable and warrants further investigation. Despite the growing diversity of the Dutch population [29], individuals with non-European migration backgrounds remain significantly underrepresented in the dermatology workforce. This is noteworthy given the importance of skin tone diversity in dermatology, as conditions manifest differently across skin types. Beyond biological differences, cultural backgrounds influence skin care practices, perceptions of disease, and health-seeking behavior, all of which can impact dermatological care [17,18,30]. Additionally, McGrath et al. found that patients of colour in the U.S. context are more likely to have a preference for a dermatologist of their ethnic background [31]. While similar research in the Dutch patient context does not yet exist, our study indicate that the number of dermatologists with specific migration backgrounds such as Turkish, Moroccan or other African, Surinamese, and Dutch Caribbean remains very low. If similar patient preferences exist in the Netherlands, this could potentially limit opportunities for physician-patient ethnic concordance in dermatology. However, further research is needed to understand whether and how such preferences play a role in the Dutch context.

Language barriers can further complicate consultations, leading to miscommunication about symptoms, misunderstandings of treatment instructions, and lower adherence to medical advice, which may contribute to delays in diagnosis or inappropriate treatments [32–34]. Additionally, some patients from minority backgrounds report feeling unheard or misunderstood by physicians, which may be linked to unconscious bias or a lack of familiarity with dermatological conditions in darker skin [8]. These challenges suggest that greater diversity in the dermatology workforce may contribute to improved care for all patient populations, though further research is needed to establish this link in the Dutch context.

The socio-economic homogeneity among dermatologists raises the question of whether this may affect patient-physician interactions with patients from lower socio-economic backgrounds. Research in other clinical settings suggests that patients with lower socio-economic status report receiving less empathy from clinicians compared to wealthier patients, potentially due to implicit biases, communication differences, and lack of shared experiences that make it difficult for physicians to understand the challenges lower-income patients face [35,36]. Our findings also indicate that physicians with at least one parent who is a registered physician are significantly more likely to become dermatologists, aligning with international research showing that children of medical doctors are overrepresented in competitive specialties due to advantages in guidance, mentorship, and professional networks [37–39]. Further research is needed to determine the specific mechanisms through which parental profession influences specialty choice and access in the Dutch context.

These patterns suggest that individuals from minority backgrounds remain underrepresented in Dutch dermatology, though our data cannot distinguish whether this reflects structural barriers in selection, differences in specialty preferences, or other factors. This is particularly relevant as dermatologists must navigate cultural differences in skincare practices, health beliefs, and communication barriers [17,30,40], all factors that may influence diagnosis and treatment. The combination of ethnic underrepresentation and socio-economic homogeneity raises questions about the factors that shape the composition of the dermatology workforce, and may have implications for care inclusivity and responsiveness for diverse patient populations.

Importantly, these dimensions of inequality do not operate in isolation. An intersectional perspective recognizes that migration background, socio-economic status, and sex interact and compound one another, creating unique barriers for individuals with multiple marginalized identities [41,42]. Research has shown that students from ethnic minority and low-income backgrounds simultaneously are significantly less likely to sustain a career path toward competitive specialties [43], and Dutch medical residents from ethnic minorities report lower perceived resemblance to their supervisors, potentially affecting their sense of belonging and career development [44]. Our own intersectional analyses confirm this within dermatology: the workforce growth between 2005 and 2023 was overwhelmingly concentrated among women without a migration background, suggesting that progress in sex diversity has not translated into broader inclusivity across ethnicity and socio-economic background.

The issue of limited diversity extends beyond the workforce into medical education. Dutch medical students have expressed dissatisfaction with their preparation for treating diverse patient populations [45]. This concern spans beyond the Netherlands; medical students in the United States [46,47] and United Kingdom [48] have identified similar deficiencies. For example, a recent study found that 87.6% of images in Dutch dermatology textbooks depict light skin, while only 12.4% show medium to dark skin tones, and merely 0.5% represent very dark skin [23]. Similar issues exist in textbooks from the United States [49–51], United Kingdom [52], Germany [53], and Scandinavia [54], and even in countries where the dominant skin color is not white, such as nations in Africa [55,56] and the Middle East [56]. Thus it is not only the dermatology workforce that lags behind in reflecting the diversity of the population it serves; the medical education system itself fails to adequately prepare future physicians to meet the needs of an increasingly diverse society [9,57,58]. Addressing these educational gaps is equally as important as improving workforce diversity to ensure that all patients, regardless of their background, receive equitable and high-quality dermatological care.

While any physician can learn about diagnosing conditions in diverse skin types, physicians with migration backgrounds may bring a deeper understanding of cultural aspects influencing diagnosis, communication, and treatment outcomes [8,9,19]. Cultural background, lived experience, and effective communication are considered important factors in dermatological care [17,18,30], especially when language barriers [32,34] and differing healthcare perceptions affect treatment adherence [17,18,30]. Further research is needed to examine the extent to which workforce diversity directly influences these aspects of care quality in the Dutch dermatological setting.

### Limitations

This study has limitations affecting the interpretation of our findings. We lacked data on both dermatology residents in training and physicians who applied for residency positions. Without transparent selection processes or a centralized application system in the Netherlands, we cannot determine whether limited diversity stems from application patterns, selection inequality, or both factors.

Additionally, the classification of “migration background” used by Statistics Netherlands is based on the country of birth of an individual and/or their parents. While this is a well-established demographic indicator, it may not fully capture the diversity of lived cultural, linguistic, or ethnic experiences within these categories. For example, a second-generation individual may have limited connection to their parents’ country of origin, while others may maintain strong cultural ties. As such, and consistent with previous research using this classification [28], this measure should be understood as a proxy rather than a direct measure of cultural or linguistic identity.

Lastly, the small size of certain demographic groups in our intersectional analysis presents another limitation. Statistics Netherlands prohibits reporting descriptive statistics for groups with <10 individuals and regression results for groups with <5 individuals. While regression analyses were performed on all groups regardless of their size, privacy regulations prevented us from publishing their outcomes for Turkish-Dutch and Moroccan-Dutch dermatologists, and young male dermatologists with an Indonesian or Other migration background. This limits our ability to fully present the unequal odds these groups of physicians face in dermatology specialization.

### Recommendations

Currently, Dutch training hospitals maintain individual selection procedures with varying transparency, criteria, and committee compositions. As in previous research [24], our study identifies persistent workforce homogeneity but cannot determine whether this stems from self-selection, inequality in selection procedures, or other mechanisms. We therefore support the call by Mulder et al. [24] for implementing a national registration system for residency training applications and selection outcomes, which would enable investigation of these underlying causes. Additionally, we propose implementing a centralized application system for residency training, combined with an evidence-based, objective and equitable recruitment and selection procedure. This could potentially promote consistency, fairness, and equal information access for all candidates. Selection processes should consider diversity as a priority, given its importance to quality of care for all patient populations [7,17,31,35,36]. As the medical student population and the younger generation of physicians shows greater ethnic diversity than the dermatology specialty [24], the potential pool of future dermatology residents in training is diverse enough to achieve a representative workforce within dermatology. It is crucial that recruitment procedures preserve this diversity throughout the specialization pathway. However, further research into the specific role of selection procedures in shaping workforce diversity is needed to inform the design of such a system.

Alongside these efforts, medical education should incorporate diverse skin representations in training materials, as the underrepresentation of darker skin tones may contribute to diagnostic challenges for minority patients.. Curriculum development should emphasize conditions across all skin tones, supported by comprehensive visual resources. Additionally, mandatory cultural competence training should be integrated into medical education and professional development, equipping dermatologists with skills to effectively serve diverse patient populations. Further research is needed to investigate whether and how workforce homogeneity may affect patient outcomes, particularly regarding diagnosis accuracy and quality of care for minority patients.

### Other information/disclaimer

This article is part of a comprehensive research project on diversity across all Dutch *BIG-register* healthcare occupations. While using consistent methodology across specialties, this paper presents unique dermatology-specific findings, including demographic data and regression analyses of physicians’ odds of specializing in dermatology. Reference group data on the (young) physician pool will appear in other papers as well, as the extensive nature of the BIG-register (covering 35 medical specialties and 10 additional healthcare occupations) necessitated dividing findings across multiple publications. It is necessary to publish these physician data in each article, so that the diversity of the dermatology workforce can be compared to the diversity of the physician workforce, both by using their respective descriptive statistics and by the results of the regression analyses, which are performed on the total physician pool and the young physician pool. This does not constitute dual publication, since the physician pool is only the reference group to which the dermatology workforce is compared.

## Supporting information

S1 Protocol Research protocol. (DOCX)

S2 Table Multivariable logistic regression. (DOCX)
