# Supplementary material for: Who becomes a dermatologist? A repeated cross-sectional study on diversity in the Dutch dermatology workforce
Source: PLoS One. 2026 Jun 12;21(6):e0350963. doi: 10.1371/journal.pone.0350963 (PMC13262814; doi:10.1371/journal.pone.0350963)
Supplement: S1 Protocol — (DOCX) [file pone.0350963.s001.docx]

**Appendix 1. Research protocol**

The results of this paper are based on calculations by [anonymized for peer review], based on non-public microdata of Statistics Netherlands (CBS). CBS Microdata are pseudonymized data which are connectable based on a pseudonymized Citizen Service Number which is the same for each individual person in each dataset. Under strict privacy regulations [1], researchers can use these data for statistical and scientific research in the secure microdata environment of Statistics Netherlands, the Remove Access environment [2]. More information about this can be found on [www.cbs.nl/microdata](http://www.cbs.nl/microdata). For more information about CBS Microdata you can email [microdata@cbs.nl](mailto:microdata@cbs.nl).

The dataset that is used for this research is the file BIGTAB [3], which is the pseudonymized BIG-register. The data in this file can be merged with other CBS datasets based on the pseudonymized Citizen Service Number of every individual (variable: RINPERSOON). RIN stands for Record Identification Number. This number forms a meaningless and dimensionless pseudonymization of the Citizen Service Number [4].

The BIG-registered healthcare professionals in the BIGTAB dataset are merged with the following files and variables:

**GBAPERSOONTAB** [4]

GBAGESLACHT: A person’s sex as registered in the BRP (Basis Registratie Personen - Basic Registration Persons).

GBAGEBOORTEJAAR: Year of birth.

GBAHERKOMSTGROEPERING: Based on the merge of this variable with the CBS reference file LANDAKTUEELREF, the migration background of each person is categorized in ten groups (variable: LANDTIENDELING): The Netherlands, Europe (excl. The Netherlands), Turkey, Morocco, Suriname, the Dutch Caribbean islands, Indonesia, Other Africa, Other Asia, and Other America and Oceania.

For this research, we created an intersectional variable, based on the combination of the variables GBAGESLACHT (sex) and LANDTIENDELING. This means that every person is categorized based on the combination of their sex and migration background at the same time.

**KINDOUDERTAB** [5]

This dataset registered the juridical parents of each person. These are not necessarily the biological parents of a person. In the dataset, the parents are classified as RINPERSOONpa (father) and RINPERSOONma (mother). Although CBS also registers the sex of each juridical parents, it was not necessary for this research to know whether somebody has a father and mother, two fathers, two mothers, or a parent with a different sex than male or female. Therefore, the sex of the parent was not included in the merge with the BIGTAB database.

The parents of the BIG-registered healthcare professionals are merged with the following files and variables:

**INPATAB** [6]

INPSECJ: Classification of a person’s socioeconomic category, based on income sources in a year. This variable was used to determine if a parent of a healthcare professional was the recipient of a social welfare income.

**VEHTAB** [7]

VEHP100HVERM: Percentile groups of the assets of private households.

**BIGTAB** [3]

BIGBEROEP: The BIG-profession of a person

BIGSPECIALISME: The BIG-specialty of a person

The addition of these variables to the dataset BIGTAB made it possible to investigate the diversity of each BIG-registered occupation and specialty in 5 different years and for two different generations in 2023, based on year of birth.

**References**

1. Statistics Netherlands (CBS). Export of data [Export van gegevens]. 2024 [Accessed 20 March 2025]. Available from: https://www.cbs.nl/nl-nl/onze-diensten/maatwerk-en-microdata/microdata-zelf-onderzoek-doen/export-van-gegevens.

2. Statistics Netherlands (CBS). Microdata: Performing your own research. Statistics Netherlands [Microdata: Zelf onderzoek doen. Centraal Bureau voor de Statistiek]. 2024 [Accessed 20 March 2025]. Available from: https://www.cbs.nl/microdata.

3. Statistics Netherlands (CBS). Bigtab: Occupations in Healthcare registrations [Bigtab: Beroepen In de Gezondheidszorg inschrijvingen]. 2024 [Accessed 20 March 2025]. Available from: https://www.cbs.nl/nl-nl/onze-diensten/maatwerk-en-microdata/microdata-zelf-onderzoek-doen/microdatabestanden/bigtab-beroepen-in-de-gezondheidszorg-inschrijvingen.

4. Statistics Netherlands (CBS). Gbapersoontab: Personal characteristics of individuals in the BRP [Gbapersoontab: Persoonskenmerken van personen in de BRP]. 2024 [Accessed 20 March 2025]. Available from: https://www.cbs.nl/nl-nl/onze-diensten/maatwerk-en-microdata/microdata-zelf-onderzoek-doen/microdatabestanden/gbapersoontab-persoonskenmerken-van-personen-in-de-brp.

5. Statistics Netherlands (CBS). Kindoudertab: individuals and their legal parents [Kindoudertab: personen en hun juridische ouders]. 2024 [Accessed 20 March 2025]. Available from: https://www.cbs.nl/nl-nl/onze-diensten/maatwerk-en-microdata/microdata-zelf-onderzoek-doen/microdatabestanden/kindoudertab-personen-en-hun-juridische-ouders.

6. Statistics Netherlands (CBS). Inpatab: Income of individuals [Inpatab: Inkomen van personen]. 2024 [Accessed 20 March 2025]. Available from: https://www.cbs.nl/nl-nl/onze-diensten/maatwerk-en-microdata/microdata-zelf-onderzoek-doen/microdatabestanden/inpatab-inkomen-van-personen.

7. Statistics Netherlands (CBS). Vehtab: Assets of households [Vehtab: Vermogens van huishoudens]. 2024 [Accessed 20 March 2025]. Available from: https://www.cbs.nl/nl-nl/onze-diensten/maatwerk-en-microdata/microdata-zelf-onderzoek-doen/microdatabestanden/vehtab-vermogens-van-huishoudens.
