# Supplementary material for: Who becomes a dermatologist? A repeated cross-sectional study on diversity in the Dutch dermatology workforce
Source: PLoS One. 2026 Jun 12;21(6):e0350963. doi: 10.1371/journal.pone.0350963 (PMC13262814; doi:10.1371/journal.pone.0350963)
Supplement: S2 Table — (DOCX) [file pone.0350963.s002.docx]

**S2. Multivariable logistic regression**

|  | **Dermatologists, born  ≥1980** | | | **Dermatologists,  all ages** | | |
| --- | --- | --- | --- | --- | --- | --- |
|  | **p-value** | **Adjusted odds ratio** | **95% C.I.** | **p-value** | **Adjusted odds ratio** | **95% C.I.** |
| **SEX** |  |  |  |  |  |  |
| Woman (ref: man) | **0,008** | **1,591** | 1,13-2,24 | 0,061 | 1,237 | 0,99-1,546 |
| **MIGRATION BACKGROUND** |  |  |  |  |  |  |
| Europe (ref: none) | 0,106 | 1,661 | 0,897-3,075 | 0,25 | 1,324 | 0,821-2,136 |
| Non-European (ref: none) | 0,123 | 1,403 | 0,912-2,158 | 0,537 | 1,109 | 0,799-1,539 |
| **PARENTAL ASSETS PERCENTILE** |  |  |  |  |  |  |
| Cat 61-80 (ref. 1-60) | 0,435 | 1,305 | 0,669-2,544 | 0,778 | 0,94 | 0,613-1,442 |
| Cat 81-100 (ref. 1-60) | **0,004** | **2,335** | 1,319-4,133 | **0,014** | **1,554** | 1,092-2,21 |
| **NUMBER OF PARENTS WHO ARE REGISTERED HEALTHCARE PROFESSIONALS** |  |  |  |  |  |  |
| 1 or 2 (ref. 0) | 0,751 | 1,051 | 0,772-1,431 | 0,524 | 1,076 | 0,859-1,348 |
