# Supplementary material for: Who becomes a dermatologist? A repeated cross-sectional study on diversity in the Dutch dermatology workforce
Source: PLoS One. 2026 Jun 12;21(6):e0350963. doi: 10.1371/journal.pone.0350963 (PMC13262814; doi:10.1371/journal.pone.0350963)
Supplement: Attachment — Submitted filename: Response to Reviewers.docx [file pone.0350963.s004.docx]

**Response to Reviewers**

**Manuscript:** PONE-D-25-63882

**Title:** *Who becomes a dermatologist? A repeated cross-sectional study on diversity in the Dutch dermatology workforce*

Dear Editor and Reviewers,

We would like to sincerely thank the Academic Editor and both reviewers for their thoughtful and constructive feedback on our manuscript. We are grateful for the positive reception of our work and greatly appreciate the valuable suggestions for improvement. We have carefully addressed each comment to the best of our ability and believe the revisions have strengthened the manuscript considerably. Below, we provide a detailed overview of all feedback received and the corresponding changes made.

**Reviewer 1 (Mahdi Salih)**

| **Comment** | **Our Response** |
| --- | --- |
| Spelling and language errors: The reviewer noted some spelling mistakes (e.g., "sociaal-economic", "microdataconducting") and suggested revision. | We have carefully proofread the entire manuscript and corrected all identified spelling and language errors. Specifically, we corrected "sociaal-economic" to "socio-economic" throughout and standardized all instances to the hyphenated form. We also corrected "medial doctors" to "medical doctors," changed "between 2005 to 2023" to "from 2005 to 2023," replaced Dutch decimal notation (commas) with English decimal points throughout the Results section, and corrected "less than" to "fewer than" where appropriate for countable quantities. |
| Intersectionality: The paper could expand on how socio-economic status and family background intersect with ethnic background to impact dermatology career progression. | We have added a new paragraph to the Discussion that explicitly addresses intersectionality. Drawing on recent literature (Samra and Hankivsky, 2021; Mosley et al., 2025; Nguyen et al., 2024), we discuss how migration background, socio-economic status, and sex do not operate as independent barriers but compound one another. We also reference a recent Dutch study among medical residents (Al-Hassany et al., 2025) and connect these findings to our own intersectional analyses, which show that workforce growth was overwhelmingly concentrated among women without a migration background. |
| Small group limitation: The lack of data for small groups (<5 individuals) in some regression models limits the analysis. Please briefly discuss how this was dealt with. | We have expanded the Methods and Limitations section to clarify that we included all groups of physicians in the regression analyses, but that we were not allowed to publish the regression results if there were fewer than 5 dermatologists in the specific group. This means that the analysis was not limited in any way (as physicians were not excluded from the regression analysis itself) but only that the outcome could not be published in the manuscript if there were fewer than 5 dermatologists with a specific background. In the Limitations section, we have changed the existing paragraph to: “Lastly, the small size of certain demographic groups in our intersectional analysis presents another limitation. Statistics Netherlands prohibits reporting descriptive statistics for groups with <10 individuals and regression results for groups with <5 individuals. While regression analyses were performed on all groups regardless of their size, privacy regulations prevented us from publishing their outcomes for Turkish-Dutch and Moroccan-Dutch dermatologists, and young male dermatologists with an Indonesian or Other migration background. This limits our ability to fully present the unequal odds these groups of physicians face in dermatology specialization.” |

**Reviewer 2 (Gabriel Lazzeri Cortez)**

| **Comment** | **Our Response** |
| --- | --- |
| Interpretive restraint in Discussion: The interpretation of associations should be more cautious. The study cannot determine whether disparities reflect structural barriers, self-selection, or other mechanisms. The Discussion occasionally moves beyond the data. | We fully agree and have systematically revised the Discussion. We replaced evaluative language (e.g., "alarming", "inaccessible") with neutral terms, reframed causal claims as hypotheses, changed "increases odds of entering dermatology" to "is associated with higher odds of being a registered dermatologist," and added calls for further research throughout where causal interpretations were implied. |
| Tempering claims about clinical impact: Claims about patient outcomes and diagnostic accuracy should be presented as areas for future research rather than direct consequences. | As part of the systematic revision described above, we have tempered all claims regarding clinical impact. Statements about misdiagnosis, care quality, and treatment outcomes are now framed as hypotheses rather than established causal links, and we have added explicit calls for further research to investigate these relationships in the Dutch context. |
| Policy recommendation: The recommendation for a centralized residency selection system should be presented more cautiously, as the study does not directly examine selection procedures. | We have revised the Recommendations section to explicitly acknowledge that, consistent with previous research by Mulder et al. (2023), our study identifies persistent workforce homogeneity but cannot determine its causes. We now first support the call by Mulder et al. for a national registration system for residency training applications and outcomes. We have retained our suggestion for a centralized application system but reframed it as a potential measure, and added a call for further research into the role of selection procedures. |
| Limitations of "migration background" classification: This classification may not fully capture lived cultural or linguistic experience. | We have added a paragraph to the Limitations section acknowledging that the Statistics Netherlands classification of migration background is based on country of birth and may not fully capture the diversity of lived cultural, linguistic, or ethnic experiences. We clarify that, consistent with previous research (Mulder et al., 2023), this measure should be understood as a proxy rather than a direct measure of cultural or linguistic identity. |
| Abstract, Study design: The study design should be explicitly stated early in the abstract. | We have revised the opening of the Methods section in the abstract to explicitly state the study design as a "nationwide, population-based, repeated cross-sectional study," in accordance with STROBE Item 1(a). |
| Abstract, Comparison group: The comparison group in the regression analysis should be more clearly defined. | We have revised the Methods section of the abstract to explicitly state that dermatologists were compared to all other registered physicians in the 2023 national healthcare professional register (BIG register). |
| Abstract, Odds ratios: The Results section should include at least one adjusted odds ratio with confidence interval. | We have added the adjusted odds ratios with 95% confidence intervals for the younger generation of physicians in the abstract:  “Multivariable regression analysis showed that being female (OR 1.609 [1.143–2.266]), having parents in the top 20% assets bracket (OR 2.251 [1.272–3.984]), or having physician parents (OR 1.326 [1.011-1.740]) were associated with higher odds of being a registered dermatologist among the younger generation of physicians.” |
| Abstract, Concluding sentence: The conclusion should be moderated to avoid implying causal effects on patient care, given the cross-sectional design. | We have revised the concluding sentence to frame the implications as exploratory, stating that the underrepresentation "may have implications for equitable patient care" and that "further research is warranted to explore the potential impact of workforce diversity on patient outcomes." |

**Academic Editor / Journal**:

| **Comment** | **Our Response** |
| --- | --- |
| Journal requirements: PLOS ONE style templates, table placement, financial disclosure, Data Availability statement, Supporting Information captions, reference check for retracted papers, and figure specifications. | We have addressed all journal requirements: formatted the manuscript according to PLOS ONE style templates, placed tables appropriately, updated the financial disclosure and funder role statement, revised the Data Availability statement, added Supporting Information captions and updated in-text citations, checked all references for retracted papers, and ensured figures meet PLOS ONE technical specifications. |
| PLOS ONE style and formatting: The manuscript must meet PLOS ONE style requirements, including file naming conventions. | We have formatted the manuscript according to PLOS ONE style templates and updated all file names accordingly. |
| Table placement: Tables should be included as part of the main manuscript. Supplementary tables should be uploaded separately as "supporting information" files. | We have placed all main tables within the manuscript and uploaded supplementary tables as separate supporting information files. |
| Financial disclosure: Update the financial disclosure statement and clarify the role of the funders in the study. | We have updated the financial disclosure statement, specifying that the funders had no role in study design, data collection and analysis, decision to publish, or preparation of the manuscript. |
| Data Availability statement: Update the Data Availability statement in the submission form. | The updated Data Availability statement is as follows:  The raw data that support the findings of this study are owned by Statistics Netherlands, and are accessible under certain conditions for statistical and scientific research. The authors of the manuscript do not own any raw data, and under Dutch law, it is not legal to publicly share these data. For further information about how to access the raw data, see the following Statistics Netherlands website: https://www.cbs.nl/en-gb/our-services/customised-services-microdata/microdata-conducting-your-own-research |
| We noted in your submission details that a portion of your manuscript may have been presented or published elsewhere. Please clarify whether this was peer-reviewed and formally published. If this work was previously peer-reviewed and published, in the cover letter please provide the reason that this work does not constitute dual publication and should be included in the current manuscript. | We have expanded on this section, which now reads as follows:  “This article is part of a comprehensive research project on diversity across all Dutch BIG-register healthcare occupations. While using consistent methodology across specialties, this paper presents unique dermatology-specific findings, including demographic data and regression analyses of physicians' odds of specializing in dermatology. Reference group data on the (young) physician pool will appear in other papers as well, as the extensive nature of the BIG-register (covering 35 medical specialties and 10 additional healthcare occupations) necessitated dividing findings across multiple publications. It is necessary to publish these physician data in each article, so that the diversity of the dermatology workforce can be compared to the diversity of the physician workforce, both by using their respective descriptive statistics and by the results of the regression analyses, which are performed on the total physician pool and the young physician pool. This does not constitute dual publication, since the physician pool is only the reference group to which the dermatology workforce is compared.” |
| Supporting Information: Include captions for supporting information files and update any in-text citations to match the Supporting Information guidelines. | We have added captions for all supporting information files and updated in-text citations to match PLOS ONE Supporting Information guidelines. |
| Reviewer-recommended publications: Evaluate whether publications recommended by reviewers are relevant and cite them if appropriate. | Neither reviewer recommended specific publications for citation. No action was required for this item. |
| Reference list: Review the reference list to ensure it is complete, correct, and check for any retracted papers. | We have reviewed the complete reference list and confirmed that no retracted papers are included. New references were added to the manuscript as a result of reviewer feedback, specifically to support the expanded discussion on intersectionality, see points above. |

We trust that these revisions adequately address all comments raised and hope the revised manuscript is now suitable for publication in PLOS ONE.

Kind regards, on behalf of all authors,

Lianne Mulder, PhD

Corresponding Author
