# Supplementary material for: Who becomes a dermatologist? A repeated cross-sectional study on diversity in the Dutch dermatology workforce
Source: PLoS One. 2026 Jun 12;21(6):e0350963. doi: 10.1371/journal.pone.0350963 (PMC13262814; doi:10.1371/journal.pone.0350963)
Supplement: Peer review history

# Peer review: Decision Letter 0

**DOI:** 10.1371/journal.pone.0350963.r001

**Authors:** Gisele Viana de Oliveira

9 Mar 2026

PONE-D-25-63882

Who Becomes a Dermatologist? A Cross-Sectional Study on Diversity in the Dutch Dermatology Workforce

PLOS One

Dear Dr. Mulder

Thank you for submitting your manuscript to PLOS ONE. After careful consideration, we feel that it has merit but does not fully meet PLOS ONE’s publication criteria as it currently stands. Therefore, we invite you to submit a revised version of the manuscript that addresses the points raised during the review process.

Please submit your revised manuscript by Apr 23 2026 11:59PM. If you will need more time than this to complete your revisions, please reply to this message or contact the journal office at plosone@plos.org. When you're ready to submit your revision, log on to https://www.editorialmanager.com/pone/ and select the 'Submissions Needing Revision' folder to locate your manuscript file.

Please include the following items when submitting your revised manuscript:

- A letter that responds to each point raised by the academic editor and reviewer(s). You should upload this letter as a separate file labeled 'Response to Reviewers'.
- A marked-up copy of your manuscript that highlights changes made to the original version. You should upload this as a separate file labeled 'Revised Manuscript with Track Changes'.
- An unmarked version of your revised paper without tracked changes. You should upload this as a separate file labeled 'Manuscript'.

-->If you would like to make changes to your financial disclosure, please include your updated statement in your cover letter. Guidelines for resubmitting your figure files are available below the reviewer comments at the end of this letter.

If applicable, we recommend that you deposit your laboratory protocols in protocols.io to enhance the reproducibility of your results. Protocols.io assigns your protocol its own identifier (DOI) so that it can be cited independently in the future. For instructions see: https://journals.plos.org/plosone/s/submission-guidelines#loc-laboratory-protocols. Additionally, PLOS ONE offers an option for publishing peer-reviewed Lab Protocol articles, which describe protocols hosted on protocols.io. Read more information on sharing protocols at https://plos.org/protocols?utm_medium=editorial-email&utm_source=authorletters&utm_campaign=protocols.

We look forward to receiving your revised manuscript.

Kind regards,

Gisele Viana de Oliveira

Academic Editor

PLOS One

Journal Requirements:

When submitting your revision, we need you to address these additional requirements.

1. Please ensure that your manuscript meets PLOS ONE's style requirements, including those for file naming. The PLOS ONE style templates can be found at

https://journals.plos.org/plosone/s/file?id=wjVg/PLOSOne_formatting_sample_main_body.pdf and

https://journals.plos.org/plosone/s/file?id=ba62/PLOSOne_formatting_sample_title_authors_affiliations.pdf.

2. Please include your tables as part of your main manuscript and remove the individual files. Please note that supplementary tables (should remain/ be uploaded) as separate "supporting information" files.

3. Thank you for stating the following financial disclosure:

“This study was made possible by the Dutch Ministry of Health, Welfare and Sports, and by a crowdfunding campaign.”

Please state what role the funders took in the study.  If the funders had no role, please state: "The funders had no role in study design, data collection and analysis, decision to publish, or preparation of the manuscript."

If this statement is not correct you must amend it as needed.

Please include this amended Role of Funder statement in your cover letter; we will change the online submission form on your behalf.

4. We noted in your submission details that a portion of your manuscript may have been presented or published elsewhere. [DETAILS AS NEEDED] Please clarify whether this [conference proceeding or publication] was peer-reviewed and formally published. If this work was previously peer-reviewed and published, in the cover letter please provide the reason that this work does not constitute dual publication and should be included in the current manuscript.

5. We note that you have indicated that there are restrictions to data sharing for this study. For studies involving human research participant data or other sensitive data, we encourage authors to share de-identified or anonymized data. However, when data cannot be publicly shared for ethical reasons, we allow authors to make their data sets available upon request. For information on unacceptable data access restrictions, please see http://journals.plos.org/plosone/s/data-availability#loc-unacceptable-data-access-restrictions.

Before we proceed with your manuscript, please address the following prompts:

a) If there are ethical or legal restrictions on sharing a de-identified data set, please explain them in detail (e.g., data contain potentially identifying or sensitive patient information, data are owned by a third-party organization, etc.) and who has imposed them (e.g., a Research Ethics Committee or Institutional Review Board, etc.). Please also provide contact information for a data access committee, ethics committee, or other institutional body to which data requests may be sent.

b) If there are no restrictions, please upload the minimal anonymized data set necessary to replicate your study findings to a stable, public repository and provide us with the relevant URLs, DOIs, or accession numbers. Please see http://www.bmj.com/content/340/bmj.c181.long for guidelines on how to de-identify and prepare clinical data for publication. For a list of recommended repositories, please see https://journals.plos.org/plosone/s/recommended-repositories. You also have the option of uploading the data as Supporting Information files, but we would recommend depositing data directly to a data repository if possible.

Please update your Data Availability statement in the submission form accordingly.

6. Please include captions for your Supporting Information files at the end of your manuscript, and update any in-text citations to match accordingly. Please see our Supporting Information guidelines for more information: http://journals.plos.org/plosone/s/supporting-information.

7. If the reviewer comments include a recommendation to cite specific previously published works, please review and evaluate these publications to determine whether they are relevant and should be cited. There is no requirement to cite these works unless the editor has indicated otherwise.

8. Please review your reference list to ensure that it is complete and correct. If you have cited papers that have been retracted, please include the rationale for doing so in the manuscript text, or remove these references and replace them with relevant current references. Any changes to the reference list should be mentioned in the rebuttal letter that accompanies your revised manuscript. If you need to cite a retracted article, indicate the article’s retracted status in the References list and also include a citation and full reference for the retraction notice.

[Note: HTML markup is below. Please do not edit.]

Reviewers' comments:

Reviewer's Responses to Questions

**Comments to the Author**

1. Is the manuscript technically sound, and do the data support the conclusions?

The manuscript must describe a technically sound piece of scientific research with data that supports the conclusions. Experiments must have been conducted rigorously, with appropriate controls, replication, and sample sizes. The conclusions must be drawn appropriately based on the data presented.

Reviewer #1: Yes

Reviewer #2: Yes

**********

2. Has the statistical analysis been performed appropriately and rigorously?

Reviewer #1: Yes

Reviewer #2: Yes

**********

3. Have the authors made all data underlying the findings in their manuscript fully available?

The PLOS Data policy requires authors to make all data underlying the findings described in their manuscript fully available without restriction, with rare exception (please refer to the Data Availability Statement in the manuscript PDF file). The data should be provided as part of the manuscript or its supporting information, or deposited to a public repository. For example, in addition to summary statistics, the data points behind means, medians and variance measures should be available. If there are restrictions on publicly sharing data—e.g. participant privacy or use of data from a third party—those must be specified.

Reviewer #1: Yes

Reviewer #2: Yes

**********

4. Is the manuscript presented in an intelligible fashion and written in standard English?

PLOS ONE does not copyedit accepted manuscripts, so the language in submitted articles must be clear, correct, and unambiguous. Any typographical or grammatical errors should be corrected at revision, so please note any specific errors here.

Reviewer #1: Yes

Reviewer #2: Yes

**********

5. Review Comments to the Author

Please use the space provided to explain your answers to the questions above. You may also include additional comments for the author, including concerns about dual publication, research ethics, or publication ethics. (Please upload your review as an attachment if it exceeds 20,000 characters)

Reviewer #1: This study assesses diversity in the Dutch dermatology workforce, focusing on sex, migration background, and socioeconomic status. Data from the BIG register (2005-2023) reveal significant increases in female dermatologists (61.7% in 2023), but ethnic diversity remains stagnant. Regression analyses show that being female, having wealthy or physician parents increases the likelihood of becoming a dermatologist.

This is a timely and relevant paper.

Minor comments:

- The paper touches on socioeconomic status and family background but could expand on intersectionality: how socioeconomic and ethnic backgrounds intersect to impact dermatology career progression.

- The lack of data for small groups (<5 individuals) in some regression models limits the analysis. Please briefly discuss how this was dealt with.

Some spelling mistakes (" recruitmenent, microdataconducting, sociaal-economic etc.). Please revise.

Reviewer #2: This manuscript addresses an important and timely issue regarding workforce diversity in Dutch dermatology. The use of nationwide registry data spanning nearly two decades is a clear strength, and the analytical approach is appropriate for the research questions posed. The topic is highly relevant for health workforce research and for ongoing discussions about equity in medical specialties. That said, several aspects of reporting and interpretation would benefit from refinement.

With respect to the abstract, it is generally clear and structured, but it could better align with reporting recommendations for observational studies (STROBE). First, the study design should be explicitly stated early in the abstract. At present, the data source and analytical approach are described, but the design is not clearly identified as a nationwide repeated cross-sectional study. Including this explicitly would immediately clarify the methodological framework. Second, the comparison group in the regression analysis should be more clearly defined. It would improve transparency to specify that dermatologists were compared with the broader physician workforce registered in the national registry. Third, the Results section of the abstract summarizes regression findings narratively; if space allows, including at least one adjusted odds ratio with its confidence interval would strengthen quantitative reporting. Finally, the concluding sentence should be moderated slightly to avoid implying causal effects on patient care, given the cross-sectional design. Framing the implications as potential or exploratory would enhance scientific precision.

Beyond the abstract, the overall methodology is sound and the statistical analyses are appropriately conducted, including assessment of multicollinearity. The use of parental asset percentiles and parental profession as socioeconomic indicators is thoughtful and yields interesting findings. However, the interpretation of these associations should be more cautious. The study identifies demographic characteristics associated with being registered as a dermatologist, but it does not include data on residency applications, acceptance rates, specialty preferences, or academic performance. As such, it cannot determine whether observed disparities reflect structural barriers, self-selection, intergenerational professional transmission, or other mechanisms. The Discussion occasionally moves beyond the data in suggesting inequitable access or exclusion; these statements should be tempered and clearly framed as hypotheses requiring further study.

Similarly, while the manuscript raises important concerns about potential implications for equitable dermatologic care, the study does not evaluate patient outcomes, diagnostic accuracy, or access to care in the Netherlands. Extrapolations from international literature are informative, but conclusions about clinical impact should be presented as areas for future research rather than direct consequences of the findings.

The policy recommendation for a centralized residency selection system is thought-provoking but currently exceeds the direct evidence provided by the analyses. As the study does not examine selection procedures themselves, these recommendations should be presented more cautiously.

Conceptually, a brief expansion on the limitations of using “migration background” based on parental country of birth would strengthen the theoretical framing, as this classification may not fully capture lived cultural or linguistic experience.

Overall, this is a valuable and well-executed study that contributes meaningful national data to an underexplored European context. The primary revisions required concern clarity of reporting in the abstract and greater interpretive restraint in the Discussion. With these adjustments, the manuscript would be considerably strengthened and well suited for publication.

**********

6. PLOS authors have the option to publish the peer review history of their article (what does this mean?). If published, this will include your full peer review and any attached files.

If you choose “no”, your identity will remain anonymous but your review may still be made public.

**Do you want your identity to be public for this peer review?**  For information about this choice, including consent withdrawal, please see our Privacy Policy.

Reviewer #1: **Yes:**  Mahdi Salih

Reviewer #2: **Yes:**  Gabriel Lazzeri Cortez

**********

[NOTE: If reviewer comments were submitted as an attachment file, they will be attached to this email and accessible via the submission site. Please log into your account, locate the manuscript record, and check for the action link "View Attachments". If this link does not appear, there are no attachment files.]

To ensure your figures meet our technical requirements, please review our figure guidelines: https://journals.plos.org/plosone/s/figures

You may also use PLOS’s free figure tool, NAAS, to help you prepare publication quality figures: https://journals.plos.org/plosone/s/figures#loc-tools-for-figure-preparation.

NAAS will assess whether your figures meet our technical requirements by comparing each figure against our figure specifications.

# Author comment: Author response to Decision Letter 1

**DOI:** 10.1371/journal.pone.0350963.r002

17 Apr 2026

Dear Editor and Reviewers,

We would like to sincerely thank the Academic Editor and both reviewers for their thoughtful and constructive feedback on our manuscript. We are grateful for the positive reception of our work and greatly appreciate the valuable suggestions for improvement. We have carefully addressed each comment to the best of our ability and believe the revisions have strengthened the manuscript considerably. Below, we provide a detailed overview of all feedback received and the corresponding changes made.

Reviewer 1 (Mahdi Salih)

Spelling and language errors: The reviewer noted some spelling mistakes (e.g., "sociaal-economic", "microdataconducting") and suggested revision. Our response: We have carefully proofread the entire manuscript and corrected all identified spelling and language errors. Specifically, we corrected "sociaal-economic" to "socio-economic" throughout and standardized all instances to the hyphenated form. We also corrected "medial doctors" to "medical doctors," changed "between 2005 to 2023" to "from 2005 to 2023," replaced Dutch decimal notation (commas) with English decimal points throughout the Results section, and corrected "less than" to "fewer than" where appropriate for countable quantities.

Intersectionality: The paper could expand on how socio-economic status and family background intersect with ethnic background to impact dermatology career progression. Our response: We have added a new paragraph to the Discussion that explicitly addresses intersectionality. Drawing on recent literature (Samra and Hankivsky, 2021; Mosley et al., 2025; Nguyen et al., 2024), we discuss how migration background, socio-economic status, and sex do not operate as independent barriers but compound one another. We also reference a recent Dutch study among medical residents (Al-Hassany et al., 2025) and connect these findings to our own intersectional analyses, which show that workforce growth was overwhelmingly concentrated among women without a migration background.

Small group limitation: The lack of data for small groups (<5 individuals) in some regression models limits the analysis. Please briefly discuss how this was dealt with. Our response: We have expanded the Methods and Limitations section to clarify that we included all groups of physicians in the regression analyses, but that we were not allowed to publish the regression results if there were fewer than 5 dermatologists in the specific group. This means that the analysis was not limited in any way (as physicians were not excluded from the regression analysis itself) but only that the outcome could not be published in the manuscript if there were fewer than 5 dermatologists with a specific background. In the Limitations section, we have changed the existing paragraph to: "Lastly, the small size of certain demographic groups in our intersectional analysis presents another limitation. Statistics Netherlands prohibits reporting descriptive statistics for groups with <10 individuals and regression results for groups with <5 individuals. While regression analyses were performed on all groups regardless of their size, privacy regulations prevented us from publishing their outcomes for Turkish-Dutch and Moroccan-Dutch dermatologists, and young male dermatologists with an Indonesian or Other migration background. This limits our ability to fully present the unequal odds these groups of physicians face in dermatology specialization."

Reviewer 2 (Gabriel Lazzeri Cortez)

Interpretive restraint in Discussion: The interpretation of associations should be more cautious. The study cannot determine whether disparities reflect structural barriers, self-selection, or other mechanisms. The Discussion occasionally moves beyond the data. Our response: We fully agree and have systematically revised the Discussion. We replaced evaluative language (e.g., "alarming", "inaccessible") with neutral terms, reframed causal claims as hypotheses, changed "increases odds of entering dermatology" to "is associated with higher odds of being a registered dermatologist," and added calls for further research throughout where causal interpretations were implied.

Tempering claims about clinical impact: Claims about patient outcomes and diagnostic accuracy should be presented as areas for future research rather than direct consequences. Our response: As part of the systematic revision described above, we have tempered all claims regarding clinical impact. Statements about misdiagnosis, care quality, and treatment outcomes are now framed as hypotheses rather than established causal links, and we have added explicit calls for further research to investigate these relationships in the Dutch context.

Policy recommendation: The recommendation for a centralized residency selection system should be presented more cautiously, as the study does not directly examine selection procedures. Our response: We have revised the Recommendations section to explicitly acknowledge that, consistent with previous research by Mulder et al. (2023), our study identifies persistent workforce homogeneity but cannot determine its causes. We now first support the call by Mulder et al. for a national registration system for residency training applications and outcomes. We have retained our suggestion for a centralized application system but reframed it as a potential measure, and added a call for further research into the role of selection procedures.

Limitations of "migration background" classification: This classification may not fully capture lived cultural or linguistic experience. Our response: We have added a paragraph to the Limitations section acknowledging that the Statistics Netherlands classification of migration background is based on country of birth and may not fully capture the diversity of lived cultural, linguistic, or ethnic experiences. We clarify that, consistent with previous research (Mulder et al., 2023), this measure should be understood as a proxy rather than a direct measure of cultural or linguistic identity.

Abstract, Study design: The study design should be explicitly stated early in the abstract. Our response: We have revised the opening of the Methods section in the abstract to explicitly state the study design as a "nationwide, population-based, repeated cross-sectional study," in accordance with STROBE Item 1(a).

Abstract, Comparison group: The comparison group in the regression analysis should be more clearly defined. Our response: We have revised the Methods section of the abstract to explicitly state that dermatologists were compared to all other registered physicians in the 2023 national healthcare professional register (BIG register).

Abstract, Odds ratios: The Results section should include at least one adjusted odds ratio with confidence interval. Our response: We have added the adjusted odds ratios with 95% confidence intervals for the younger generation of physicians in the abstract: "Multivariable regression analysis showed that being female (OR 1.609 [1.143–2.266]), having parents in the top 20% assets bracket (OR 2.251 [1.272–3.984]), or having physician parents (OR 1.326 [1.011-1.740]) were associated with higher odds of being a registered dermatologist among the younger generation of physicians."

Abstract, Concluding sentence: The conclusion should be moderated to avoid implying causal effects on patient care, given the cross-sectional design. Our response: We have revised the concluding sentence to frame the implications as exploratory, stating that the underrepresentation "may have implications for equitable patient care" and that "further research is warranted to explore the potential impact of workforce diversity on patient outcomes."

Academic Editor / Journal

Journal requirements: PLOS ONE style templates, table placement, financial disclosure, Data Availability statement, Supporting Information captions, reference check for retracted papers, and figure specifications. Our response: We have addressed all journal requirements: formatted the manuscript according to PLOS ONE style templates, placed tables appropriately, updated the financial disclosure and funder role statement, revised the Data Availability statement, added Supporting Information captions and updated in-text citations, checked all references for retracted papers, and ensured figures meet PLOS ONE technical specifications.

PLOS ONE style and formatting: The manuscript must meet PLOS ONE style requirements, including file naming conventions. Our response: We have formatted the manuscript according to PLOS ONE style templates and updated all file names accordingly.

Table placement: Tables should be included as part of the main manuscript. Supplementary tables should be uploaded separately as "supporting information" files. Our response: We have placed all main tables within the manuscript and uploaded supplementary tables as separate supporting information files.

Financial disclosure: Update the financial disclosure statement and clarify the role of the funders in the study. Our response: We have updated the financial disclosure statement, specifying that the funders had no role in study design, data collection and analysis, decision to publish, or preparation of the manuscript.

Data Availability statement: Update the Data Availability statement in the submission form. Our response: The updated Data Availability statement is as follows: The raw data that support the findings of this study are owned by Statistics Netherlands, and are accessible under certain conditions for statistical and scientific research. The authors of the manuscript do not own any raw data, and under Dutch law, it is not legal to publicly share these data. For further information about how to access the raw data, see the following Statistics Netherlands website: https://www.cbs.nl/en-gb/our-services/customised-services-microdata/microdata-conducting-your-own-research

Prior presentation or publication: We noted in your submission details that a portion of your manuscript may have been presented or published elsewhere. Please clarify whether this was peer-reviewed and formally published. If this work was previously peer-reviewed and published, in the cover letter please provide the reason that this work does not constitute dual publication and should be included in the current manuscript. Our response: We have expanded on this section, which now reads as follows: "This article is part of a comprehensive research project on diversity across all Dutch BIG-register healthcare occupations. While using consistent methodology across specialties, this paper presents unique dermatology-specific findings, including demographic data and regression analyses of physicians' odds of specializing in dermatology. Reference group data on the (young) physician pool will appear in other papers as well, as the extensive nature of the BIG-register (covering 35 medical specialties and 10 additional healthcare occupations) necessitated dividing findings across multiple publications. It is necessary to publish these physician data in each article, so that the diversity of the dermatology workforce can be compared to the diversity of the physician workforce, both by using their respective descriptive statistics and by the results of the regression analyses, which are performed on the total physician pool and the young physician pool. This does not constitute dual publication, since the physician pool is only the reference group to which the dermatology workforce is compared."

Supporting Information: Include captions for supporting information files and update any in-text citations to match the Supporting Information guidelines. Our response: We have added captions for all supporting information files and updated in-text citations to match PLOS ONE Supporting Information guidelines.

Reviewer-recommended publications: Evaluate whether publications recommended by reviewers are relevant and cite them if appropriate. Our response: Neither reviewer recommended specific publications for citation. No action was required for this item.

Reference list: Review the reference list to ensure it is complete, correct, and check for any retracted papers. Our response: We have reviewed the complete reference list and confirmed that no retracted papers are included. New references were added to the manuscript as a result of reviewer feedback, specifically to support the expanded discussion on intersectionality, see points above.

We trust that these revisions adequately address all comments raised and hope the revised manuscript is now suitable for publication in PLOS ONE.

Kind regards, on behalf of all authors,

Lianne Mulder, PhD

Corresponding Author

Attachment Submitted filename: Response to Reviewers.docx

# Editor report: Decision Letter 1

**DOI:** 10.1371/journal.pone.0350963.r003

**Authors:** Gisele Viana de Oliveira

20 May 2026

Who becomes a dermatologist? A repeated cross-sectional study on diversity in the Dutch dermatology workforce

PONE-D-25-63882R1

Dear Dr. Mulder

We’re pleased to inform you that your manuscript has been judged scientifically suitable for publication and will be formally accepted for publication once it meets all outstanding technical requirements.

Within one week, you’ll receive an e-mail detailing the required amendments. When these have been addressed, you’ll receive a formal acceptance letter and your manuscript will be scheduled for publication.

An invoice will be generated when your article is formally accepted. Please note, if your institution has a publishing partnership with PLOS and your article meets the relevant criteria, all or part of your publication costs will be covered. Please make sure your user information is up-to-date by logging into Editorial Manager at Editorial Manager® and clicking the ‘Update My Information' link at the top of the page. For questions related to billing, please contact billing support.

If your institution or institutions have a press office, please notify them about your upcoming paper to help maximize its impact. If they’ll be preparing press materials, please inform our press team as soon as possible -- no later than 48 hours after receiving the formal acceptance. Your manuscript will remain under strict press embargo until 2 pm Eastern Time on the date of publication. For more information, please contact onepress@plos.org.

Kind regards,

Gisele Viana de Oliveira

Academic Editor

PLOS One

Additional Editor Comments (optional):

The authors carefully revised the manuscript to address all reviewer and editorial comments. They corrected language and formatting issues throughout the paper, clarified methodological points, and expanded the discussion on intersectionality and demographic disparities. They also added clearer explanations about limitations related to small subgroup analyses and the use of “migration background” as a proxy measure.

Discussion and Conclusions were rewritten in a more cautious and balanced way. Claims that could imply causation were softened, clinical implications were reframed as hypotheses requiring further research, and policy recommendations were presented more carefully.

The abstract was strengthened by explicitly stating the study design, clarifying the comparison group, and including adjusted odds ratios with confidence intervals.

Finally, the authors fully updated the manuscript to comply with PLOS ONE requirements, including formatting, tables, supplementary files, financial disclosure, data availability statements, figure specifications, and reference checks. They also clarified concerns regarding overlap with related publications from the same broader research project.

# Editor report: Acceptance letter

**DOI:** 10.1371/journal.pone.0350963.r004

**Authors:** Gisele Viana de Oliveira

PONE-D-25-63882R1

PLOS One

Dear Dr. Mulder,

I'm pleased to inform you that your manuscript has been deemed suitable for publication in PLOS One. Congratulations! Your manuscript is now being handed over to our production team.

At this stage, our production department will prepare your paper for publication. This includes ensuring the following:

* All references, tables, and figures are properly cited

* All relevant supporting information is included in the manuscript submission,

* There are no issues that prevent the paper from being properly typeset

You will receive further instructions from the production team, including instructions on how to review your proof when it is ready. Please keep in mind that we are working through a large volume of accepted articles, so please give us a few days to review your paper and let you know the next and final steps.

Lastly, if your institution or institutions have a press office, please let them know about your upcoming paper now to help maximize its impact. If they'll be preparing press materials, please inform our press team within the next 48 hours. Your manuscript will remain under strict press embargo until 2 pm Eastern Time on the date of publication. For more information, please contact onepress@plos.org.

You will receive an invoice from PLOS for your publication fee after your manuscript has reached the completed accept phase. If you receive an email requesting payment before acceptance or for any other service, this may be a phishing scheme. Learn how to identify phishing emails and protect your accounts at https://explore.plos.org/phishing.

If we can help with anything else, please email us at customercare@plos.org.

Thank you for submitting your work to PLOS ONE and supporting open access.

Kind regards,

PLOS ONE Editorial Office Staff

on behalf of

Dr. Gisele Viana de Oliveira

Academic Editor

PLOS One
